# Surgical Mask to Prevent Influenza Transmission in Households: A Cluster Randomized Trial

**DOI:** 10.1371/journal.pone.0013998

**Published:** 2010-11-17

**Authors:** Laetitia Canini, Laurent Andréoletti, Pascal Ferrari, Romina D'Angelo, Thierry Blanchon, Magali Lemaitre, Laurent Filleul, Jean-Pierre Ferry, Michel Desmaizieres, Serge Smadja, Alain-Jacques Valleron, Fabrice Carrat

**Affiliations:** 1 UMR-S 707, UPMC – Paris 6, Paris, France; 2 U707, Inserm, Paris, France; 3 Laboratoire de Virologie Médicale et Moléculaire, Centre Hospitalier Universitaire de Reims, Reims, France; 4 EA-4303, Faculté de Médecine, Reims, France; 5 Unité de Santé Publique de L'hôpital Saint-Antoine, Assistance Publique Hôpitaux de Paris, Paris, France; 6 Cire Aquitaine, Institut de Veille Sanitaire, Bordeaux, France; 7 Cabinet Médical, Audincourt, France; 8 Urgences Médicales de Paris, Paris, France; 9 SOS Médecins, Paris, France; Cochrane Acute Respiratory Infections Group, Italy

## Abstract

**Background:**

Facemasks and respirators have been stockpiled during pandemic preparedness. However, data on their effectiveness for limiting transmission are scarce. We evaluated the effectiveness of facemask use by index cases for limiting influenza transmission by large droplets produced during coughing in households.

**Methodology and Principal Findings:**

A cluster randomized intervention trial was conducted in France during the 2008–2009 influenza season. Households were recruited during a medical visit of a household member with a positive rapid influenza A test and symptoms lasting less than 48 hours. Households were randomized either to the mask or control group for 7 days. In the intervention arm, the index case had to wear a surgical mask from the medical visit and for a period of 5 days. The trial was initially intended to include 372 households but was prematurely interrupted after the inclusion of 105 households (306 contacts) following the advice of an independent steering committee. We used generalized estimating equations to test the association between the intervention and the proportion of household contacts who developed an influenza-like illness during the 7 days following the inclusion. Influenza-like illness was reported in 24/148 (16.2%) of the contacts in the intervention arm and in 25/158 (15.8%) of the contacts in the control arm and the difference between arms was 0.40% (95%CI: −10% to 11%, P = 1.00). We observed a good adherence to the intervention. In various sensitivity analyses, we did not identify any trend in the results suggesting effectiveness of facemasks.

**Conclusion:**

This study should be interpreted with caution since the lack of statistical power prevents us to draw formal conclusion regarding effectiveness of facemasks in the context of a seasonal epidemic.

**Trial Registration:**

clinicaltrials.gov NCT00774774

## Introduction

Influenza virus is responsible for annual epidemics worldwide and causes a significant public health burden. Influenza virus is transmitted by direct contact with infected individuals, exposure to virus-contaminated objects (fomites), and inhalation of infectious aerosols [1]. The threat of a severe H5N1 pandemic caused by avian influenza and the recent worldwide spreading of influenza H1N1v have renewed interests in nonpharmaceutical interventions for limiting influenza transmission. Hand sanitizers, facemasks and respirators have been stockpiled during pandemic preparedness and are currently recommended in several countries. However, data on their effectiveness for limiting transmission are scarce. Five randomized trials evaluating facemasks and hand hygiene with different designs and objectives have been recently published [2], [3], [4], [5], [6]. Three of these trials were conducted in families and did not show significant improvements in their primary analyses in intervention groups versus the control groups [2], [3], [4]. However, in these trials, secondary analysis suggested that intervention using face masks could have a significant effectiveness if implemented rapidly from illness onset or providing a good adherence to the intervention [7].

In this study, we evaluated the effectiveness of surgical facemasks for limiting influenza transmission by large droplets produced during coughing. A clustered design was justified since the intervention was randomly assigned to a household member and outcomes were measured in their household contacts.

## Methods

The protocol for this trial and supporting CONSORT checklist are available as supporting information; see Checklist S1 and Protocol S1.

### Population description, eligibility and enrollment

We conducted a cluster-randomized controlled trial in which households were randomly allocated to a surgical mask arm (intervention) or a control (non-intervention) arm. The mask had to be worn by the index case only. The intervention was targeted at the household level, and the outcomes were measured at the individual level in household subjects. The households were selected by 62 general practitioners, who were volunteers to participate to the study.

The study was conducted in 3 French regions (Ile de France, Aquitaine and Franche-Comté) during the 2008–09 influenza season period – defined as the period when the national incidence of influenza-like illness (ILI) reported on the French national influenza surveillance Sentinelles system was above a calculated threshold [8].

Households of size 3 to 8 were eligible. Households were recruited by general practitioners (GP) when one member (the index patient) aged over 5 years old had a medical visit with the GP for symptoms lasting less than 48 hours, combining temperature over 37.8°C and cough, and a positive rapid test for influenza A (Quick View® Influenza A+B Test, Quidel Corp., San Diego, CA, USA). The index patient had to be *a priori* the first and unique illness case in the household and be affiliated to the French national health insurance. Households were not eligible if the index patient was treated for asthma or chronic obstructive pulmonary disease or was hospitalized. Written informed consent was obtained from the index patient before rapid testing. Proxy written consent from parents or legal guardians was obtained for persons 17 years or younger, with additional written assent from those 13 to 17 years of age and eventually from those 7 to 13 years old. The GP graded each symptom and sign exhibited by the index patient from 0 = none, 1 = mild, 2 = moderate, and 3 = intense. In each household, a referent adult member accepted the follow-up responsibility for the trial. The referent member received a tympanic thermometer with instructions to safely take the temperature of every household member with this device at day 0, 3, and 6 or in case of new symptoms. The referent accepted to complete a questionnaire on a daily basis and during a period of 21 days, with details on symptoms, health care use, quality of life and social activities of all household members. Instructions were also given to the referent member to maintain blinding during the telephone interview.

### Ethics statement

The study protocol was approved by the ethics committee Comité de Protection des Personnes Ile de France XI and was registered with ClinicalTrials.gov (Identifier NCT00774774).

### Randomization

Households were randomized in a 1∶1 ratio either to the mask or control group. Randomization was stratified according to age of the index patient (<15 years, ≥15 years) [9] and the French administrative region. Randomization lists were prepared by a biostatistician, according to the block randomized method, with blocks of size 2 to 6.

Randomization lists were generated by a computerized program. Randomization was performed centrally by the GP after written consent on an interactive voice response system dedicated to the study.

### Intervention

In case of randomization in the mask group, thirty masks were given immediately and a demonstration for proper use was given by the GP. Surgery masks with earloops, 3 plys, anti fog (AEROKYN®, LCH medical products, Paris, France) were used for adults and children over 10 years of age. Children facemasks (Face Mask KC47127, Kimberly-Clark®, Dallas, TX, USA) were used for index patients aged 5 to 10 years old. The masks had to be worn from the medical visit and for a period of 5 days, each time another household member was in the same room or in a confined place (e.g. in a car). The index patient did not have to wear the mask at night. The masks had to be changed every 3 hours, or if they were damaged, and disposed of in closed plastic bags. In both groups, the index patient was encouraged to sleep alone in his/her room. In the control group, no intervention was applied. Seven days from inclusion, the referent household member was contacted by phone by a trained investigator. The interview was assisted with a computer program and was run in two steps: a first step during which the investigator was blinded to the treatment arm and the referent member was solicited to report on symptoms (including temperature), treatment, medical and social outcomes, for every household member; a second unblinded step during which the arm was revealed to the investigator and the referent member was solicited to report on mask use and observance in the index patient, in case of randomization in the intervention group.

The main objective was to assess the decrease of secondary illness in household contact in the mask group *vs.* the control group. We also focused on the tolerance and feasibility of wearing masks.

### Sample Size

The size of the study assumed an analysis at the individual level, a proportion of 24% of secondary illness in household contacts and an intra-cluster correlation of 0.29 [10]. We expected an absolute decrease of 10% of the attack rate for the contact subjects (a relative decrease of 42%); this value was considered as clinically relevant. The mean size of households with more than 3 members is 3.8 [11]. Therefore, 372 households representing 1042 contacts and a total of 1414 subjects were necessary to obtain a power of 90%.

### Definition of outcomes

The primary endpoint was the proportion of household contacts who developed an ILI during the 7 days following inclusion. A temperature over 37.8°C with cough or sore throat was used as primary clinical case-definition [12]. This definition has been shown to be specific to influenza infection during seasonal epidemics [4], but of limited sensitivity. We therefore also used a more sensitive case-definition (hereafter referred to as the sensitive ILI definition) based on a temperature over 37.8°C or at least two of the following: sore throat, cough, runny nose, or fatigue [4]. *Post hoc* analyses were conducted by taking into account the time between symptoms onset in the index patient and allocation to intervention or by considering ILI that occurred after a minimum time lag after allocation to intervention [3]. We also studied whether occurrence of an ILI in the intervention arm was associated with adherence parameters (such as duration of wearing a mask). Finally, we explored a cluster level efficacy outcome, the proportion of households with 1 or more secondary illness in household contacts. Other analyses were conducted regarding adverse reactions due to mask-wearing, the number of days of mask-wearing and the number of masks worn.

### Statistical analysis

All analyses were done on an intent-to-treat basis. The rule missing equal failure was applied for missing outcome values. To compare study outcomes between arms, we used cluster-specific method because households rather than patients were randomized. The main outcomes were compared at the individual contact level considering the index case age stratification and a within household correlation. We estimated 95% CIs of proportions by using a cluster bootstrap technique with 1000 resamples [13]. We used an alternating logistic regression model with an exchangeable log odds ratio to test the multivariate-adjusted association between the intervention and the outcomes and to identify other predictors associated with the outcomes [14]. The exchangeable log odds ratio is a measure of within household correlation and should be interpreted as a ratio of the odds of a contact to be a secondary case when another contact in the household is a secondary case to the odds of a contact to be a secondary case when another contact is not a secondary case. We used forced-entry methods to include the allocated group and factors that may not have been well balanced between arms at baseline, while other potential predictors were included based on a P-value<0.20 in univariate analysis and were selected using a backward procedure. Baseline signs and symptoms of index patients were dichotomized in two levels, none or mild versus moderate or intense. The Fisher's exact test was used to compare proportions. To determine the compliance factors associated with ILI among contacts tests in the intervention group, we performed bivariate analyses using Wilcoxon 2-sample test. For all analyses, a P-value of 0.05 was considered as statistically significant. Analyses were performed using SAS v9.1.3 (Sas Institute Inc, Cary, NC, USA)

### Early stopping

The trial was initially intended to be conducted during a single influenza season, during winter 2008–09. In early March 2009, due to a mild and short influenza season, approximately thirty percent of the expected number of households had been included, and the scientific committee of the trial was solicited to decide on whether or not the accrual period should be extended over the subsequent influenza season. The decision was to conduct the trial over the next season. However, as the new H1N1v emerged and because the French national preparedness included mass distribution of surgical facemasks in households, methodological and ethical concerns about the possibility to pursue the trial occurred. In June 2009, the scientific committee requested advice from an independent steering committee. An unblinded preliminary analysis was presented to the independent committee during a closed meeting excluding investigators of the trial and the sponsor, and the decision was made to stop the trial.

## Results

An influenza epidemic caused by seasonal A/Brisbane/10/2007 (H3N2)-like was announced the 15th December 2008, by the influenza surveillance systems in France and ended the 22nd February 2009 [15]. For practical reasons, inclusions in the trial started just after the national Christmas holidays.

**Figure 1:**
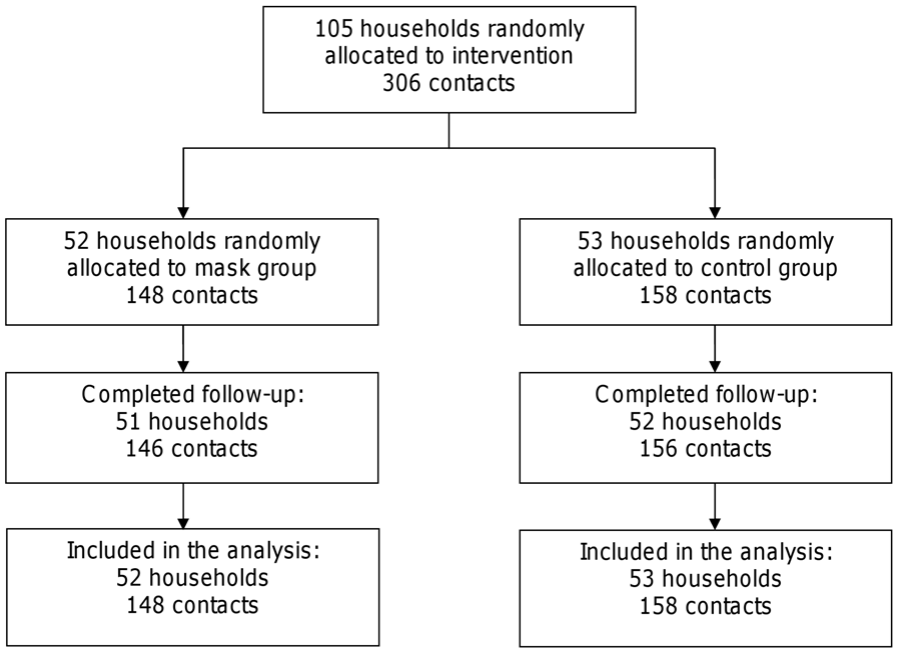
Flow diagram.

Between January 5^th^ 2009 to February 16^th^ 2009, 32 general practitioners recruited 105 households, which represented 148 contacts in the intervention arm and 158 in the control arm. Two households were lost to follow-up, one in each arm (Figure 1), they were considered in the analysis.

The characteristics of the index patients and the household contacts in the two arms were overall similar (Table 1): 35 (33%) of index patients were children under 15 years of age, 50 (48%) were female, and influenza symptoms were well balanced between arms. The most frequent symptoms (except cough which was one of the inclusion criteria) were fatigue, headache, myalgia, runny nose/sneezing, sore throat, lacrimation and earache. The mean size of the household was 3.9±1.0. Differences were however observed concerning the proportion of index patients who were smokers and the proportion of household contacts less than 15 years in the intervention arm.

**Table 1:** Characteristics of the index cases and household contacts.

	Intervention arm	Control arm
**Index case – number**	**52**	**53**
Age (years) – mean ± SD	25±16	28±16
Age<15 years – n (%)	19 (37)	16 (30)
Sex ratio (M/F)	26/26	29/24
Vaccinated – n (%)	0 (0)	2 (4)
Current smoker: yes – n (%)	15 (29)	2 (4)
Time between symptoms onset and allocation to intervention (hours)		
≤6 – n (%)	0 (0)	1 (2)
7–12 – n (%)	8 (15)	8 (15)
13–18 – n (%)	13 (25)	10 (19)
19–24 – n (%)	8 (15)	14 (26)
25–36 – n (%)	11 (21)	10 (19)
37–48 – n (%)	10 (19)	9 (17)
Not available – n (%)	2 (4)	1 (2)
Symptoms of the index case*		
Fatigue – n (%)	50 (96)	48 (92)
Headache – n (%)	37 (73)	40 (77)
Myalgia – n (%)	38 (73)	38 (73)
Runny nose/sneezing – n (%)	37 (71)	36 (69)
Sore throat – n (%)	24 (46)	21 (40)
Lacrimation – n (%)	18 (35)	22 (42)
Earache – n (%)	4 (8)	5 (10)
Body temperature (°C) - mean ± SD	38.2±0.8	38.3±0.8
Number of household contacts - mean ± SD	2.8±1.1	3.0±1.0
**Household contacts – number**	**148**	**158**
Age (years) – mean ± SD	29±19	25±17
Age<15 years – n (%)	41 (28)	62 (39)
Sex ratio (M/F)	73/75	79/79
Vaccinated – n (%)	14 (9)	6 (4)
Current smoker: yes – n (%)	24 (16)	20 (13)

*number (%) of patients exhibiting moderate or intense symptoms.

The primary analysis was unconclusive: ILI was reported in 24/148 (16.2%) of the contacts in the intervention arm and in 25/158 (15.8%) of the contacts in the control arm. The difference -of ILI attack rate between the intervention arm and the control arm was 0.40% (95%CI: −10% to 11%, P = 1.00). ILI among contacts occurred more frequently when moderate or intense sore throat or runny nose or elevated temperature were reported in the index patient, and when the household contact was aged less than 15 years old and was female (Table 2). The multivariate adjusted odds-ratio (OR) for intervention arm *vs.* control arm was 0.95 (95%CI: 0.44 to 2.05, P = 0.90). The common log-odds ratio was 3.66 (95%CI 1.53 to 8.73, P = 0.0035). Using the sensitive ILI definition did not modify the findings: 42/148 (28.4%) of ILI were observed among the contacts in the intervention arm and 42/158 (26.6%) in the control arm (difference: −1.8%, 95%CI −12% to 14%; P = 0.80) and the multivariate adjusted OR for intervention arm *vs.* control arm was 0.99 (95%CI: 0.51 to 1.93, p = 0.97).

**Table 2:** Predictors of ILI among household contacts.

	Odds-Ratio	95% CI	P-value
**Index characteristics**			
Intervention arm: yes vs. no*	0.95	0.44–2.05	0.90
Current smoker: yes vs. no*	1.83	0.56–5.97	0.32
Runny nose/sneezing: intense or moderate vs. mild or none	4.61	1.44–14.8	0.010
Sore throat: intense or moderate vs. mild or none	2.52	1.15–5.53	0.021
Body temperature: per °C increase	2.04	1.07–3.89	0.030
**Contacts characteristics**			
Age<15 years: yes vs. no*	2.01	1.10–3.66	0.023
Contact's sex: Male vs. Female	0.40	0.21–0.73	0.0031

*Entry was forced for these variables in the multivariate model.

When the analysis was limited to households where the index patient was allocated to intervention less than 24 hours from symptoms onset, ILI occurred in 15 of 83 (18.1%) contacts in the intervention arm *vs.* 17 of 108 (15.7%) contacts in the control arm (difference 2.3%, 95%CI −12% to 16%; P = 0.70).

When the analysis was limited to an event that appeared more than 24 hours after inclusion, 12 ILI (9.2%) were reported in 130 contacts in the intervention arm *vs.* 13 (9.4%) in 138 contacts in the control arm (difference −0.19%, 95%CI −9.2% to 8.2%; P = 1.00). Using the sensitive ILI definition did not modify the findings.

The proportion of households with one or more secondary illness in contacts did not differ between arms. The proportion was 15/52 (29%) in the intervention arm and 18/53 (34%) in the control arm (difference −5.1%, 95%CI −23% to 13%; P = 0.67) using the primary clinical case-definition. The proportion was 22/52 (42%) in the intervention arm and 27/53 (51%) in the control arm (difference −8.6%, 95%CI −28% to 10%; P = 0.44) using the sensitive ILI definition.

In the intervention arm, the index patients reported wearing a total of 11±7.2 masks during 4.0±1.6 days with an average use of 2.5±1.3 masks per day and a duration of use of 3.7±2.7 hours a day. The adherence to mask-wearing was not associated with the ILI among contacts (Table 3).

**Table 3:** Adherence to mask use.

	ILI among contacts (n = 22)	No ILI among contacts (n = 124)	P value
Total number of masks used – mean ± SD	9.4±6.9	11.1±7.1	0.31
Number of days the mask was worn – mean ± SD	4.0±1.6	4.1±1.5	0.87
Number of masks used each day– mean ± SD	2.3±1.2	2.6±1.2	0.17
Duration of mask wearing by day (in hours) – mean ± SD	3.2±2.2	4.0±2.7	0.098

One household (2 contacts) with missing follow-up information was not included in the calculation.

Thirty-eight (75%) patients from the intervention arm reported discomfort with mask use (Table 4). The three main causes of discomfort were warmth (45%), respiratory difficulties (33%) and humidity (33%). Children wearing children facemasks reported feeling pain more frequently (3/12) than other participants wearing adult facemasks (1/39) (p = 0.036). No difference was detected concerning the other cause of discomfort depending on the facemask type.

**Table 4:** Discomfort due to mask use.

Reported problem	Children mask (n = 12)	Adult mask (n = 39)*	P value
Warmth - n (%)	5 (42)	18 (46)	1.00
Respiratory difficulties - n (%)	2 (17)	15 (39)	0.29
Humidity - n (%)	3 (25)	14 (36)	0.73
Did not like being seen with the mask - n (%)	3 (25)	12 (31)	1.00
Irritation - n (%)	2 (17)	5 (13)	0.66
Pain - n (%)	3 (25)	1 (2.6)	0.036

One index-patient with missing follow-up information was not included in the calculation.

## Discussion

We did not show any significant difference in ILI proportion among household contacts between the intervention arm and the control arm. Our study was clearly underpowered due to its premature termination. The inclusion of 105 households instead of 372 led to 38% power for detecting the hypothesized difference of 10%. There was no laboratory verification of ILI self-reports and asymptomatic or subclinical infections may have been missed in addition to including non-influenza events - altogether this may have contributed to diminish the chance to identify a significant effect of face masks. As a consequence of the lack of power, the lower bound of the 95% confidence interval of the multivariate adjusted odds-ratio was 0.44 meaning that we cannot formally exclude that our trial could have missed a substantial face masks effectiveness; i.e. a relative reduction of the ILI attack rate of up to 56%. However we did not identify any trend in the results or during the numerous secondary analyses suggesting that inclusion of the planned sample size could result in significant differences. We observed a good adherence to intervention. In 34 of 51 (66%) households of the intervention arm with follow-up information, masks were worn more than 80% of the anticipated duration. We did not identify any difference in adherence to mask use between households with secondary illnesses and households without secondary illness. Therefore we do not believe that a limited adherence may explain our findings, contrarily to what has been reported in other studies where less than 50% of the participants were adherent to the intervention [3], [6].

The analysis of other trials testing the efficacy of facemasks use in households did not show a significant decrease of the secondary illness rate in their primary intent-to-treat analyses [7]. Only one trial evaluated masks worn by the index patient. In a secondary analysis of this trial, household contacts of the intervention arm including hand hygiene and face masks had lower rates of secondary illness than the control arm if interventions were applied quickly [3]. These results indicated that a substantial proportion of influenza infection could be transmitted by other routes than large droplets, and in particular via fomites or contaminated surfaces. However, in this study, no additional benefit was observed when facemask was added to hand hygiene by comparison with hand hygiene alone. Our findings are consistent with these results, suggesting a low effectiveness, if any, of facemasks when used alone to limit influenza transmission in a closed-setting.

We identified that younger contacts (≤15 years) were more at risk of ILI than adults which is consistent with results from other studies [9], [16], [17]. The fact that women were more at risk for ILI than men may have been due to the fact that women are more often the caregiver in the households [17]. Finally, the fact that we identified an association between symptoms and influenza transmission is logical since patients with the more intense symptoms are those who shed the highest viral load [18]. In conclusion, although our findings did not suggest that face masks could prevent transmission of influenza in households, the lack of statistical power prevents us to draw a formal conclusion as to exclude that face masks could nevertheless have a substantial effect. Therefore our study should be interpreted cautiously as providing additional data to other trials realized in the context of seasonal epidemics.

### Registering clinical trials

This study was registered with ClinicalTrials.gov, number NCT: 00774774

## Supporting Information

Checklist S1 CONSORT Checklist
(0.22 MB DOC)

Protocol S1 Trial Protocol
(0.03 MB DOC)
