# Supplementary material for: Surgical Mask to Prevent Influenza Transmission in Households: A Cluster Randomized Trial
Source: PLoS One. 2010 Nov 17;5(11):e13998. doi: 10.1371/journal.pone.0013998 (PMC2984432; doi:10.1371/journal.pone.0013998)
Supplement: Protocol S1 — Trial Protocol (0.03 MB DOC) [file pone.0013998.s002.doc]

|  | **Odds-Ratio** | **95% CI** | **P-value** |
| --- | --- | --- | --- |
| **Index characteristics** |  |  |  |
| Intervention arm: yes vs no* | 0.95 | 0.44-2.05 | 0.90 |
| Current smoker: yes vs no* | 1.83 | 0.56-5.97 | 0.32 |
| Runny nose/sneezing:  intense or moderate vs mild or none | 4.61 | 1.44-14.8 | 0.010 |
| Sore throat:  intense or moderate vs mild or none | 2.52 | 1.15-5.53 | 0.021 |
| Body temperature: per °C increase | 2.04 | 1.07-3.89 | 0.030 |
| **Contacts characteristics** |  |  |  |
| Age < 15 years: yes vs no* | 2.01 | 1.10-3.66 | 0.023 |
| Contact’s sex: Male vs Female | 0.40 | 0.21-0.73 | 0.0031 |

* Entry was forced for these variables in the multivariate model
